# Dioxygen dissociation over man-made system at room temperature to form the active α-oxygen for methane oxidation

**DOI:** 10.1126/sciadv.aaz9776

**Published:** 2020-05-13

**Authors:** Edyta Tabor, Jiri Dedecek, Kinga Mlekodaj, Zdenek Sobalik, Prokopis C. Andrikopoulos, Stepan Sklenak

**Affiliations:** J. Heyrovský Institute of Physical Chemistry, The Czech Academy of Sciences, Dolejskova 3, 18223 Prague, Czech Republic.

## Abstract

Activation of dioxygen attracts enormous attention due to its potential for utilization of methane and applications in other selective oxidation reactions. We report a cleavage of dioxygen at room temperature over distant binuclear Fe(II) species stabilized in an aluminosilicate matrix. A pair of formed distant α-oxygen species [i.e., (Fe(IV)═O)^2+^] exhibits unique oxidation properties reflected in an outstanding activity in the oxidation of methane to methanol at room temperature. Designing a man-made system that mimicks the enzyme functionality in the dioxygen activation using both a different mechanism and structure of the active site represents a breakthrough in catalysis. Our system has an enormous practical importance as a potential industrial catalyst for methane utilization because (i) the Fe(II)/Fe(IV) cycle is reversible, (ii) the active Fe centers are stable under the reaction conditions, and (iii) methanol can be released to gas phase without the necessity of water or water-organic medium extraction.

## Teaser

Highly active and stable inorganic material for dissociation of dioxygen and methane oxidation to methanol was developed.

## INTRODUCTION

Activation of dioxygen is a basic enzymatic reaction of living organism (*1*). Conversely, mimicking this process over artificial inorganic systems represents a great challenge, which has not yet been successfully managed (*2*). Moreover, the activation of dioxygen gained significance as a possible key for the usage of methane. Methane as the main component of natural gas became abundant because of the development of the shale gas technology. Nevertheless, until now, productions of energy (electricity and heat) and hydrogen represent the main utilization of the methane production. Therefore, the transformation of methane to liquid products representing energy carriers and chemical production platforms is in high demand. The application of the Fischer-Tropsch process for this purpose is energetically, technologically, and investably very demanding. Conversely, the selective oxidation of methane to methanol is suggested to be an encouraging way of a methane-to-liquid transformation (*3*–*6*). However, only the selective oxidation of methane by dioxygen is economically feasible and represents a promising system of the utilization of methane with an enormous industrial impact. Conversely, the selective oxidation of methane by H_2_O_2_ and N_2_O is rather only of academic interest as the price of both oxidants is higher than that of methanol (*7*–*9*). However, it is generally accepted that Fe-zeolites can be oxidized by nitrous oxide but not by molecular oxygen to yield the unique active α-oxygen atoms that activate the methane C─H bond at ambient temperature and pressure (*10*).

In this field, the recently presented low-temperature selective oxidation of methane by molecular oxygen over Cu clusters in zeolites represents a substantial step forward (*3*–*5*); however, this route has several drawbacks: (i) the requirement of high/low-temperature cycles, (ii) an extremely low yield in the catalytic regime, and (iii) the necessity of water or water-organic medium extraction of methoxy groups strongly bound to the catalyst. These inconveniences make these systems rather inapplicable (*11*, *12*). Here, we report a previously unidentified system based on distant binuclear divalent iron centers stabilized in the matrix of a zeolite, which is a crystalline microporous aluminosilicate. This system is able to activate dioxygen by a new mechanism—a direct dissociation followed by the formation of a pair of the distant α-oxygen atoms. This α-oxygen species with special oxidation properties is so active that it can oxidize methane to methanol at room temperature. Moreover, the redox cycle (i.e., the oxidation by dioxygen and the subsequent reduction by methane) over these structures can be repeated as methanol is removed from the sample by an evacuation at 200°C without the necessity of water or water-organic medium extraction. This result therefore opens a new possible route to the development of previously unknown selective oxidation processes for methane utilization.

Panov *et al*. (*13*) reported highly reactive atomic oxygen for the Fe-ZSM-5 zeolite. This α-oxygen on Fe is defined as the active oxygen species formed by the N_2_O oxidation of Fe and capable of oxidizing H_2_, CO (*13*), benzene (*14*, *15*), and methane (*13*, *16*). The structure of the isolated [Fe(IV)═O]^2+^ or [Fe(III)-O^−●^]^2+^ species is suggested to be balanced by the negative charges of two AlO_4_^−^ tetrahedra located in the ring of the zeolite framework, which creates an extra-framework cationic site for divalent cations (*6*, *17*). Recently, Fe(II) cations stabilized in the framework of the ferrierite zeolite and forming distant binuclear cationic structures were reported to notable facilitate the abstraction of the oxygen atom from N_2_O to yield the highly active α-oxygen on the metal cation (*17*, *18*). The Fe(II) cations forming these species are located in two adjacent extra-framework cationic β sites (*17*, *18*). The calculated distance of the two Fe(II) cations is 7.4 Å (*17*, *18*). In contrast to isolated Fe(II) cations, the binuclear Fe(II) species can arrange a four-electron reaction. Moreover, these structures in ferrierite exhibit a resemblance in geometry and the oxidation state with iron active sites of methane monooxygenases; however, the distance between the two Fe cations in the enzymes is less than half of that in Fe-ferrierite (*19*, *20*). This raises a question if distant binuclear Fe(II) structures are able to cleave dioxygen and form a pair of the distant α-oxygen atoms on the two Fe cations. Subsequently, the two α-oxygen atoms can oxidize methane. To answer this question, periodic density functional theory (DFT) calculations on the reactivity of the distant binuclear Fe(II) sites were performed followed by a verification of the computational results by Mössbauer and Fourier transform infrared (FTIR) spectroscopies and stoichiometric reaction tests.

## RESULTS

**Fig. 1:**
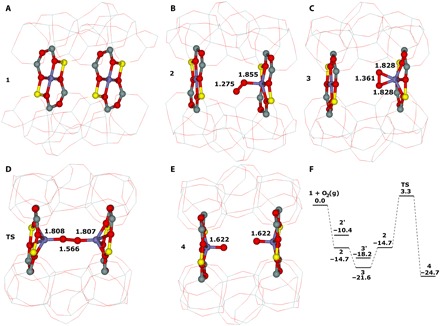
Optimized structures. (**A**) The two adjacent β sites of Fe-ferrierite **1** after molecular dynamics (MD) simulations. (**B**) The monodentate Fe OO_mono_…Fe complex **2** formed in the two adjacent β sites. (**C**) The bidentate Fe OO_bi_…Fe complex **3** formed in the two adjacent β sites. (**D**) The transition state **TS** created in the two adjacent β sites. (**E**) The Fe═O O═Fe product **4** created in the two adjacent β sites. The distances are in angstroms. Silicon atoms are in gray, oxygen atoms in red, aluminum atoms in yellow, and iron atoms in blue. Schematic energy profile (in kilocalorie per mole). (**F**) The formation of the Fe═O O═Fe product.

The periodic DFT calculations were performed for the computational model with two Fe(II) cations in two adjacent β sites of ferrierite as in our prior studies on the N_2_O decomposition (*17*) and the oxidation of Me(II) by N_2_O (*18*). An O_2_(g) molecule that is in a triplet state adsorbs on one of the Fe(II) cations to yield a [Fe OO_mono_…Fe]′ monodentate complex **2′** with the O_2_ moiety in a triplet state. **2′** either undergoes a spin crossover to give a [Fe OO_mono_…Fe] monodentate complex **2**, which has the O_2_ moiety in a singlet state, or rearranges its structure to form a [Fe OO_bi_…Fe]′ bidentate complex **3′**. In the latter case, a spin change occurs and a [Fe OO_bi_…Fe] bidentate complex **3** with the O_2_ moiety in a singlet state is yielded. The oxidation occurs from the most stable bidentate complex **3**, which rearranges to the less stable monodentate complex **2**. The adsorbed O_2_ moiety of **2** is better positioned for the interaction with the other Fe(II) located in the adjacent β site. Subsequently, dioxygen is cleaved via a [Fe-O-O-Fe] transition state **TS** to yield a [Fe═O O═Fe] complex **4** in a concerted manner. Both Fe in **4** are oxidized to form a pair of the distant α-oxygen atoms. The reaction energy of the reaction from **1** + O_2_(g) to give **4** is −24.7 kcal/mol. The calculated barrier of the cleavage of dioxygen is 24.9 kcal/mol, indicating that the oxidation should be facile but substantially more sluggish than the oxidation of the same Fe(II)-ferrierite by N_2_O [i.e., the barrier of 14.5 kcal/mol (*18*)]. The optimized structures of **1**, **2**, **3**, **TS**, and **4** and the corresponding calculated adsorption energies are shown in Fig. 1.

It should be noted that, although the distant binuclear Fe(II) sites resemble the active site of methane monooxygenases, both the oxidized structure and the mechanism of the oxygen activation are quite unique. In contrast to the creation of the bridging oxo structure of the active site of the enzyme, a pair of the distant α-oxygen atoms is formed by the cleavage of dioxygen. This is caused by a much larger distance between the two Fe cations and a relative rigidity of the zeolite framework.

The mechanism of the selective oxidation of methane by the α-oxygen on Fe in a zeolite to form methanol has already been computationally studied using periodic DFT (*21*). A further investigation of this mechanism is out of the scope of this study.

A Fe-ferrierite sample with distant binuclear Fe(II) cationic sites was prepared to test the computational predictions. The formation of distant binuclear Fe(II) cationic centers in zeolites requires fulfilling three conditions. (i) The presence of two adjacent 6 or 8 rings able to form cationic sites for bare divalent cations. The two adjacent rings must face each other. (ii) Each of the two rings has to contain two Al atoms (i.e., four Al atoms in total). The two rings can therefore form two adjacent cationic sites for bare divalent cations. (iii) The occupation of the two adjacent cationic sites by two Fe(II) cations.

The Si/Al ratio of the investigated ferrierite sample (*17*, *22*, *23*) is 8.6, meaning that there are, in average, 3.75 Al atoms per unit cell. The prior study showed that the concentration of Al pairs in the β site is high (50% of all the Al atoms) (*22*), so ca. 94% of the 6 rings of the β site can accommodate bare divalent cations. Therefore, at least 88% (0.94**2) of the β sites are able to form binuclear Fe(II) structures (*17*). A low Fe/Al ratio of 0.04 was used for Fe-ferrierite to guarantee the exclusive presence of atomically dispersed Fe(II) sites without the formation of oxo species. Fe cations were introduced using the acetylacetonate route to assure the creation of binuclear Fe(II) sites. This procedure guarantees the formation of binuclear Fe(II) structures even at the lowest Fe loadings (*17*, *24*, *25*).

**Fig. 2:**
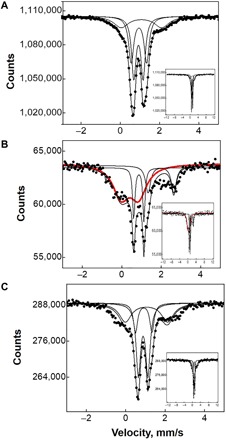
Mössbauer spectra and their fits of ^57^Fe-ferrierite. (**A**) Mössbauer spectra after an evacuation at 450°C for 3 hours. (**B**) An evacuation at 450°C for 3 hours, then an interaction with O_2_ (10^5^ Pa) at room temperature for 40 min, and after that, a desorption of O_2_ at 200°C for 5 min. The curve in red represents the α-oxygens. (**C**) An evacuation at 450°C for 3 hours and then an interaction with O_2_ (10^5^ Pa) at room temperature for 40 min, and subsequently, a desorption of O_2_ at 200°C for 5 min, and after that, an interaction with CH_4_ (10^5^ Pa) at room temperature for 40 min, and subsequently, an evacuation of CH_4_ at 200°C for 5 min. The insets show the corresponding full-range spectra.

Mössbauer spectroscopy was used to analyze the oxidation state and the coordination of the Fe species in ^57^Fe-ferrierite. Mössbauer spectra are shown in Fig. 2, and the corresponding Mössbauer parameters are listed in Table 1.

**Table 1:** Mössbauer parameters and spectral contributions of ^57^Fe-ferrierite.

**^57^Fe-FER**	**Isomer shift**	**Quadrupole splitting**	**Rel.**	**Fe species**
Fe/Al 0.04	mm/s	mm/s	%	
Evacuated*	0.93	0.46	33	Fe(II)
	0.98	0.72	40	Fe(II)
	1.02	2.06	27	Fe(II)
+O_2_†	0.89	0.51	10	Fe(II)
	1.60	1.32	18	Fe(II)
	0.38	0.78	72	Fe(III)-O^−●^
+O_2_/CH_4_‡	0.98	0.49	39	Fe(II)
	0.90	0.83	31	Fe(II)
	1.05	2.14	30	Fe(II)

*An evacuation at 450°C for 3 hours.

†An evacuation at 450°C for 3 hours, then an interaction with O_2_ at room temperature for 40 min, and after that, an O_2_ desorption at 200°C for 5 min.

‡An evacuation at 450°C for 3 hours, then an interaction with O_2_ at room temperature for 40 min, and consequently, a desorption of O_2_ at 200°C for 5 min, and after that, an interaction with CH_4_ at room temperature for 40 min, and subsequently, an evacuation of CH_4_ at 200°C for 5 min.

The results reveal that exclusively atomically dispersed Fe(II) is present in the sample after the evacuation at 450°C. The observed Mössbauer parameters evidence bare Fe(II) in the α and β cationic sites (Fig. 2A). The interaction of Fe-ferrierite with dioxygen at room temperature (followed by an evacuation) results in its oxidation reflected in the signals (Fig. 2B) attributed to atomically dispersed [Fe(IV)═O]^2+^ species [equivalent to [Fe(III)-O^−●^]^2+^ of Panov *et al.* (*26*)]. This result proves the oxidation of Fe(II) cations and suggests the formation of a pair of the distant α-oxygen atoms over the distant binuclear Fe(II) sites. The residual Fe(II) cations correspond to isolated Fe(II) in the α and β cationic sites. Both the Mössbauer spectra and parameters are similar to those obtained for the same Fe-ferrierite after an interaction with N_2_O (*18*). The α-oxygen was originally defined by its capability to oxidize H_2_, CO (*13*), benzene (*14*, *15*), and methane (*13*, *16*). Therefore, the oxidation of these molecules represents an unquestionable proof of the α-oxygen. Methane was selected for this purpose because of the enormous economic potential of the oxidation of methane to methanol. The interaction of the oxidized Fe-ferrierite with methane followed by an evacuation result in the regeneration of the initial state with the exclusive presence of the bare Fe(II) cations accommodated in the cationic sites (Fig. 2C). This result clearly evidences the formation of the α-oxygen. Moreover, in contrast to (i) the α-oxygen on isolated Fe cations and (ii) Cu-oxo species, the formation of volatile products released by an evacuation only at 200°C indicates a protonation of the formed methoxy groups and a subsequent desorption of the oxidation products from the active sites without the necessity of water or water-organic medium extraction. Similar results were recently reported for the α-oxygen formed by the decomposition of N_2_O over distant binuclear cationic sites in ferrierite (*18*). It should be noted that this result represents a notable improvement in comparison with not only (i) the selective oxidation of methane by N_2_O over isolated Fe cations in zeolites but also (ii) methane oxidation by dioxygen over Cu-zeolites. Water aid for the protonation of methoxy groups and for the desorption of methanol from the active Cu sites is essential for the Cu-zeolites. However, water vapor, on the other hand, destroys or poisons the Cu active sites formed in the Cu-zeolites.

**Fig. 3:**
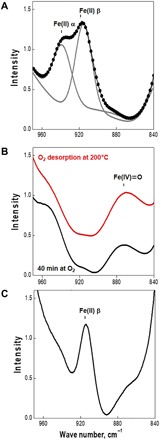
FTIR spectra of Fe-ferrierite. (**A**) FTIR spectra after an evacuation at 450°C for 3 hours. The measured data correspond to the dots, while the gray curves represent the deconvoluted profiles. (**B**) An evacuation at 450°C for 3 hours and then an interaction with O_2_ (10^5^ Pa) at room temperature for 40 min (black curve), and after that, a desorption of O_2_ at 200°C for 5 min (red curve). (**C**) An evacuation at 450°C for 3 hours and then an interaction with O_2_ (10^5^ Pa) at room temperature for 40 min, and subsequently, a desorption of O_2_ at 200°C for 5 min, and after that, an interaction with CH_4_ (10^5^ Pa) at room temperature for 40 min, and subsequently, an evacuation of CH_4_ at 200°C for 5 min.

FTIR spectroscopy of the shifted antisymmetric T-O-T stretching vibrations of the lattice induced by binding Fe cations to the framework oxygens represents a complementary tool for the analysis of the state and location of the Fe cations in zeolite frameworks and their interactions with guest molecules. FTIR spectrum of the dehydrated Fe-ferrierite (Fig. 3A) confirms the presence of bare Fe(II) in the α and β (predominating) cationic sites reflected in the bands at 938 and 918 cm^−1^, respectively.

The interaction with dioxygen at room temperature results in (i) the disappearance of the bands attributed to the bare Fe(II) cations accommodated in the β site and (ii) the formation of a new band at around 880 cm^−1^ (Fig. 3B). An analogous band has already been reported for Fe-ferrierite after the oxidation by N_2_O (*27*, *28*). This band is assigned to the α-oxygen, and it corresponds to the shifted antisymmetric T-O-T stretching vibrations of the zeolite framework, reflecting the increasing interaction of the [Fe═O]^2+^ complex with the zeolite framework. It should be noted that the maximum formation of the α-oxygen at room temperature was reached within 40 min (Fig. 3B). The subsequent interaction of the oxidized Fe-ferrierite with methane at room temperature followed by an evacuation at 200°C leads to the regeneration of Fe(II) in the β cationic site (Fig. 3C). At the most, a partial restauration of the band at 938 cm^−1^ of the bare Fe(II) cations accommodated in the α cationic site may (i) reflect the adsorption of some species (methanol and other products of the oxidation of methane) at this site; (ii) result from a different shape of the high-energy edge of the window of the shifted antisymmetric T-O-T stretching vibrations of the zeolite framework, which can result from, for example, the adsorption of guest molecules on other than Fe(II) sites; and (iii) be caused by a migration of Fe(II) cations from the α centers to the more stable (*17*) β cationic sites. In conclusion, both the FTIR and Mössbauer spectroscopy results confirm the oxidation of methane by dioxygen at room temperature. Moreover, the evacuation of the protonated products of the oxidation of methane at 200°C indicates that the products of methane oxidation are volatile species, and therefore, there is no need of water or water-organic medium extraction of the products. On the other hand, this opens a possibility that in contrast to systems with isolated α-oxygens, the methane oxidation does not stop at the formation of methoxy groups but proceeds further not only to methanol and other products of the selective oxidation but also to CO_2_ and water as the main oxidation products. Such a total oxidation activity can, in opposition to the highly positive role of the protonation of the methoxy groups to methanol, represent a substantial drawback in the development of the system for selective oxidation of methane to methanol and other valuable products.

**Fig. 4:**
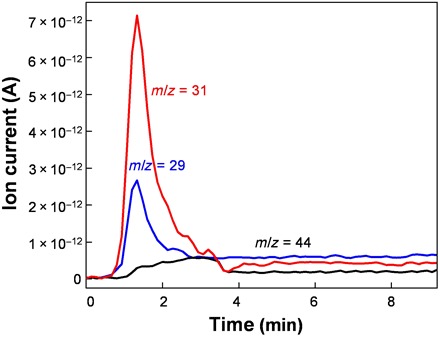
The mass spectrometry results for Fe-ferrierite. Time dependence of the intensity of the signals of ion currents reflecting the products of the oxidation of methane by the α-oxygen formed on Fe-ferrierite during the first oxidation cycle monitored by mass spectrometry (MS). The signal with *m*/*z* = 31 relates to methanol, that with *m*/*z* = 44 links with CO_2_, and that with *m*/*z* = 29 corresponds to methanol and other possible oxidation products (i.e., formaldehyde, formic acid, and dimethyl ether).

A titration of the α-oxygen by methane was performed by a through-flow experiment to analyze (i) the nature of the products of the oxidation of methane, (ii) the reactivity of pairs of the distant α-oxygen atoms, and (iii) the release of the products of the oxidation not only from the active sites but also from the zeolite crystal to the gas phase. It should be noted that a re-adsorption of methanol and other oxidation products on other adsorption centers as, for example, Fe cations in the α cationic sites, Brønsted protonic sites (Al-OH-Si), and terminal silanol groups (Si-OH) can occur. The Fe-ferrierite sample was activated in argon (3 hours at 450°C) and subsequently oxidized by dioxygen at room temperature. Then, the created α-oxygen was titrated by methane at room temperature. The formation of methanol was confirmed by the presence of the two main signals with mass/charge ratio (*m*/*z*) = 31 and 29 (Fig. 4).

Since other possible oxidation products of methane (i.e., formaldehyde, formic acid, and dimethyl ether) are also represented by the signal with *m*/*z* = 29, the signal with *m*/*z* = 31 was exclusively used to quantify the formation of methanol (Fig. 4). The signal with (*m*/*z* = 44) corresponding to CO_2_ was monitored as well (Fig. 4). After the stabilization of the mass spectrometry (MS) signal, a second oxidation cycle was performed also at room temperature. The sample was purged with Ar, and then the sample reacted in an O_2_ flow, and after purging with Ar, the oxidized sample interacted with CH_4_ under the same conditions as in the first cycle (details are in Materials and Methods).

The MS results (Fig. 4) confirm (i) the selective oxidation of methane on the distant binuclear Fe(II) sites in ferrierite already at room temperature and (ii) a protonation of the formed methoxy groups to yield methanol. The formation methanol at room temperature undoubtedly evidences splitting dioxygen to give the highly active α-oxygen species stabilized over distant binuclear Fe(II) structures accommodated in the ferrierite matrix at room temperature. The dissociation of dioxygen can be regarded as a proof of the distant binuclear Fe(II) sites as at least two Fe(II) cations are required to cooperate in the four-electron process. Therefore, the sites that are able to selectively oxidize methane to methanol at room temperature are exactly the same sites that activate dioxygen by its splitting to a pair of the distant α-oxygen atoms.

The yields of the produced methanol (i) per gram of the Fe(II) zeolite, (ii) per mole of the Fe(II) bare cations, and (iii) per mole of the Fe(II) bare cations accommodated in the β sites calculated from the results of the MS experiments for the first and second cycles are concluded in Table 2. The high molar yield of methanol per mole of the Fe(II) cations located in the β sites during the first cycle (0.63 mol_CH3OH_/mol_Fe_) evidences that methanol is the main product of the selective oxidation of methane. This value represents the lower bound of methanol formed per active site since also isolated Fe(II) cations can be present in the β sites. In the second cycle, the presence of methanol and other oxidation products was observed in the MS analysis. The yields of methanol obtained in the first and second cycles are concluded in Table 2. This evidences that distant binuclear Fe(II) bare cations in FER can be regenerated after the selective oxidation of methane even at room temperature, and therefore, a repeated dissociation of dioxygen followed by the selective oxidation of methane can be performed. However, both the signals with *m*/*z* = 29 and 31 are less intense compared the first oxidation cycle, and the methanol yield decreased by ca. 30% (Table 2). This can be explained by the blocking of some binuclear Fe(II) sites by the adsorption of less volatile oxidation products as formaldehyde and formic acid at room temperature. The formation of the latter oxidation products justifies a lower than 100% yield of methanol formed per Fe(II) in the β site in the first cycle. It should be noted that the full recovery of the Fe(II) cations in the β sites occurred only after an evacuation at 200°C according to the FTIR experiments (Fig. 3C).

**Table 2:** Yields of methanol from the MS experiments.

	**Yield of** **CH_3_OH [μmol/** **g_cat_]**	**CH_3_OH/Fe** **[mol/mol]**	**CH_3_OH/Fe_β_** **[mol/mol]**
First cycle	75	0.44	0.63
Second cycle	52	0.30	0.44

## DISCUSSION

This work describes fully inorganic aluminosilicate matrix with distant binuclear Fe(II) structures (the Fe…Fe distance is ca. 7.4 Å), which only remotely resemble the iron active sites of methane monooxygenases (the Fe…Fe distance is less than ca. 4 Å). The distant binuclear Fe(II) cations are able to cleave dioxygen at room temperature and form a pair of the distant α-oxygen atoms. Both the mechanism of the oxygen activation and the oxidized structures notably differ from those of methane monooxygenases. These α-oxygen atoms exhibit special oxidation properties and are able to selectively oxidize methane mainly to methanol at room temperature. The formation of methanol results in the reconstruction of the distant binuclear Fe(II) species, allowing a new splitting of dioxygen to occur followed by a subsequent selective oxidation of methane. This opens a possibility for the development of an active and stable system for the selective oxidation of methane to methanol.

Our results show that methane can be oxidized by dioxygen over distant binuclear Fe(II) species stabilized in an aluminosilicate matrix. This outcome indicates a breakthrough in the development of the technology of the transformation of methane to liquid products representing energy carriers and chemical production platforms. Nevertheless, the application in the chemical industry requires a further development of a long-term stable system with a high activity in the methane conversion. The regeneration of the active sites by an evacuation at 200°C and especially only a 30% decrease of the methanol yield in the second cycle at room temperature represent only good starting points in this endeavor. Attention should be focused on the development of zeolite matrices, allowing the accommodation of higher amounts of distant binuclear species and, furthermore, to reach their maximum population by Fe(II) to obtain the supreme activity in the transformation of methane.

## MATERIALS AND METHODS

### Sample preparation and treatment

Commercially supplied ferrierite Si/Al 8.6 (Tosoh Corporation, Japan) represents a well-developed material (high crystallinity, exclusively framework Al, and adequate adsorption properties) (*18*, *22*, *23*, *27*, *29*, *30*). The parent zeolite was ion-exchanged (3 × 24 hours, room temperature, 100 ml of solution per 1 g of zeolite) with 1 M NH_4_NO_3_, washed by distilled water, and dried at room temperature to prepare the NH_4_ form of ferrierite.

The Fe(II)-ferrierite catalyst with Fe/Al 0.04 was prepared by the impregnation of NH_4_-ferrierite by acetylacetone solution of FeCl_3_. Then, the sample was evacuated for 1 hour at 100°C and consequently for 3 hours at 350°C. After cooling to room temperature, the sample was washed with distilled water and calcined overnight under air flow at 420°C. The preparation procedure is described in details elsewhere (*17*, *24*, *25*, *27*). The ^57^Fe(II)-ferrierite zeolite with Fe/Al 0.04 for Mössbauer spectroscopy measurements was prepared by the same procedure using isotopically enriched ^57^FeCl_3_.

### Mössbauer spectroscopy

Mössbauer spectroscopic measurements were performed using self-supporting pellets of ^57^Fe-ferrierite. The Mössbauer spectra were recorded at ambient temperature under dynamic vacuum (10^−3^ Pa) after the following: (i) an evacuation at 450°C for 3 hours (Fig. 2A); (ii) an evacuation at 450°C for 3 hours, then an interaction with O_2_ (10^5^ Pa; purity, 99.999) at room temperature for 40 min, and after that, an O_2_ desorption at 200°C for 5 min (Fig. 2B); and (iii) an evacuation at 450°C for 3 hours, then an interaction with O_2_ (10^5^ Pa; purity, 99.999) at room temperature for 40 min, and consequently, a desorption of O_2_ at 200°C for 5 min, and after that, an interaction with CH_4_ (10^5^ Pa; purity, 99.999) at room temperature for 40 min, and subsequently, an evacuation of CH_4_ at 200°C for 5 min (Fig. 2C).

The source of γ-rays was ^57^Co in a rhodium matrix, and the α-Fe foil was used as a reference. The spectra were deconvoluted into Lorentzian-shaped components using the MossWinn software [(*31*)]. The individual components were characterized by the isomer shift describing the oxidation state of the Fe species and the quadrupole splitting related to the Fe coordination. The assignment of the Mössbauer parameters to the Fe species in ^57^Fe-ferrierite was based on our prior studies (*29*, *30*, *32*).

### Infrared spectroscopy

Infrared spectra were recorded using a Nicolet 6700 FTIR spectrometer (resolution 2 cm^−1^, 32 scans/min) equipped with a liquid nitrogen–cooled wide band mercury cadmium telluride (MCT-B) detector and KBr windows. The samples in a form of self-supporting pellets (10 mg cm^−2^) were placed in the cuvette, allowing evacuation and dosing of gases. FTIR spectra of the Fe-ferrierite samples were recorded after the following treatments: (i) an evacuation at 450°C for 3 hours (Fig. 3A); (ii) an evacuation at 450°C for 3 hours and then an interaction with O_2_ (10^5^ Pa; purity, 99.999) at room temperature for 40 min (black curve in Fig. 3B); (iii) an evacuation at 450°C for 3 hours, then an interaction with O_2_ (10^5^ Pa; purity, 99.999) at room temperature for 40 min, followed by a desorption of O_2_ at 200°C for 5 min (red curve in Fig. 3B); (iv) an evacuation at 450°C for 3 hours, then an interaction with O_2_ (10^5^ Pa, purity 99.999) at room temperature for 40 min, followed by an O_2_ desorption at 200°C for 5 min, and subsequently, an interaction with CH_4_ (10^5^ Pa; purity, 99.999) at room temperature for 40 min followed by an evacuation at 200°C for 5 min (Fig. 3C).

### Mass spectrometry

The products of the titration of the α-oxygen by methane in the through-flow experiment were monitored by MS (Balzers QMG 421 C quadrupole mass spectrometer). The sample placed in the quartz reactor was activated in a flow of Ar (20 ml/min) for 3 hours at 450°C. Subsequently, the sample was treated in a flow of O_2_ (40 ml/min; purity, 99.999) at room temperature for 1 hour followed by a purging with Ar (20 ml/min) for 5 min. Last, the oxidized sample was left to interact with CH_4_ (30 ml/min; purity, 99.999) at room temperature. After the stabilization of the MS signal, a second oxidation cycle was also performed at room temperature under the same conditions as the first cycle. The sample was purged with Ar for 5 min, and then it was left in an O_2_ flow for 30 min and subsequently purged with Ar for 5 min; following that, the oxidized sample interacted with CH_4_ at room temperature. The signal with *m*/*z* = 31 relates to methanol, that with *m*/*z* = 44 links with CO_2_, and that with *m*/*z* = 29 corresponds to methanol and other possible oxidation products (i.e., formaldehyde, formic acid, and dimethyl ether). Therefore, the quantitative analysis is based only on the signal with *m*/*z* = 31, which represents exclusively methanol. A calibration using injections of a set of 10 different amounts of methanol (0.2, 0.5, 1, 5, 12, 32, 48, 61, 81, and 94 μmol) covering the entire estimated range of yields for the Fe-ferrierite sample used (Fe/Al = 0.04) was performed. The calibration data for methanol were subsequently used to quantify the yield of methanol (Fig. 4).

### Computational models

One model was used for the Fe(II) cations. The model has *P*1 symmetry and features a super cell composed of two unit cells along the *c* dimension (i.e., *a* = 18.651, *b* = 14.173, and *c* = 14.808 Å). The model contains four Al/Si substitutions forming two β sites with the four Al atoms located in the T2 sites of the two adjacent 6 rings [only the β site with both Al in T2 was found in the ferrierite framework of this ferrierite sample (*22*)], accommodating two Fe(II) cations. The model serves to investigate the possible activity of binuclear Fe(II) structures. The starting structure was generated from the experimental orthorhombic structure of ferrierite determined by neutron diffraction (*33*).

### Electronic structure calculations

Periodic DFT calculations were carried out by using the Vienna Ab initio Simulation Package (VASP) code (*34*–*36*). The high-spin electron configuration d5↑d1↓ was used for the Fe species accommodated in the zeolite. The Kohn-Sham equations were solved variationally in a plane-wave basis set using the projector-augmented wave method of Blöchl, as adapted by Kresse and Joubert (*37*). The exchange-correlation energy was described by the Perdew-Burke-Ernzerhof (PBE) generalized gradient approximation functional. Brillouin zone sampling was restricted to the Γ point. A plane-wave cutoff of 600 eV and the density-dependent energy correction (dDsC) dispersion correction (*38*, *39*) were used for geometry optimizations, and a smaller cutoff of 400 eV and the DFT-D2 method (*40*) were used for the molecular dynamics (MD) simulations.

### Molecular dynamics

MD simulations were carried out on the model. The MD computations used the exact Hellmann-Feynman forces acting on atoms and applied the statistics of canonical ensemble to the motion of the atomic nuclei by using the Verlet velocity algorithm to integrate Newton’s equations of motion. The time step for the integration of the equations of motion was 1 fs. The simulations were run for 10,000 fs at 400 K. Visual inspection of the structures along the MD trajectories showed that the duration of the MD simulations was long enough, because it included both the rearrangements of the local structures of the ferrierite framework (up to ca. 2000 fs) and a long period (ca. 8000 fs) when the system fluctuated around the equilibrium and “snapshots” were collected and optimized. Similar time lengths were used for MD simulations of cationic sites in zeolites (*17*, *18*, *23*). The MD simulations serve to obtain the rearranged local structures [details are provided in our prior studies (*17*, *23*)]. The rearrangement can be monitored by visual inspection. No physical quantity is derived from the MD trajectories. The structures of 20 distinct snapshots collected at 500, 1000, 1500, … 10,000 fs of the MD simulations were optimized for the computational models.

### Geometry optimizations

The collected snapshots were optimized. The atomic positions were optimized at constant volume by using a conjugate-gradient algorithm minimization of energies and forces, whereas the lattice parameters were fixed at their experimental values.
